# Molecular Survey of Hemotropic *Mycoplasma* spp. and *Bartonella* spp. in Coatis (*Nasua nasua*) from Central-Western Brazil

**DOI:** 10.3390/pathogens12040538

**Published:** 2023-03-30

**Authors:** Lívia Perles, Wanessa Teixeira Gomes Barreto, Filipe Martins Santos, Leidiane Lima Duarte, Gabriel Carvalho de Macedo, Darci Moraes Barros-Battesti, Heitor Miraglia Herrera, Rosangela Zacarias Machado, Marcos Rogério André

**Affiliations:** 1 Vector-Borne Bioagents Laboratory (VBBL), Department of Pathology, Reproduction and One Health, School of Agricultural and Veterinarian Sciences, São Paulo State University (Unesp), Via de Acesso Prof. Paulo Donato Castellane, s/n, Zona Rural, Jaboticabal 14884-900, Brazil; 2 Post-Graduation of Ecology and Conservation, Mato Grosso do Sul Federal University, Campo Grande 13471-410, Brazil; 3 Laboratory of Parasitic Biology, Environmental Sciences and Farming Sustainability, Dom Bosco Catholic University, Campo Grande 13471-410, Brazil; 4 Department of Preventive Veterinary Medicine and Animal Science, School of Veterinary Medicine, University of São Paulo, São Paulo 05508-220, Brazil

**Keywords:** hemotropic *Mycoplasma* spp., *Amblyomma* spp., bartonellae, *Neotrichodectes pallidus*, midwestern Brazil

## Abstract

Even though previous works showed molecular evidence of hemotropic *Mycoplasma* spp. (hemoplasmas) in ring-tailed coatis (*Nasua nasua*) from Brazil, *Bartonella* sp. has not been reported in these mammals so far. The present study aimed to detect the above-mentioned agents in coatis’ blood and associated ectoparasites, assessing the association between these infections and red blood parameters. Between March 2018 and January 2019, coati (n = 97) blood samples, *Amblyomma* sp. ticks (2242 individual ticks, resulting in 265 pools), and *Neotrichodectes pallidus* louse (n = 59) were collected in forested urban areas from midwestern Brazil. DNA extracted from coatis’ blood, and ectoparasite samples were submitted to quantitative PCR (qPCR) (16S rRNA) and conventional PCR (cPCR) (16S rRNA and 23S rRNA) for hemoplasmas and qPCR (*nuoG* gene) and culturing (only blood) for *Bartonella* spp. Two different hemoplasma genotypes were detected in blood samples: 71% coatis positive for myc1 and 17% positive for myc2. While 10% of ticks were positive for hemoplasmas (myc1), no louse was positive. The estimated bacterial load of hemoplasmas showed no association with anemia indicators. All coatis were negative for *Bartonella* sp. in qPCR assay and culturing, albeit two *Amblyomma* sp. larvae pools, and 2 *A. dubitatum* nymph pools were positive in the qPCR. The present work showed a high occurrence of hemoplasmas, with two distinct hemoplasma genotypes, in coatis from forested urban areas in midwestern Brazil.

## 1. Introduction

Hemotropic mycoplasmas (hemoplasmas) (Mycoplasmatales: Mycoplasmataceae) are obligate epi-erythrocytic bacteria that might be transmitted by aggressive interactions between animals [1,2]. Previous works showed a high occurrence of hemoplasmas among raccoons (*Procyon lotor*) in the USA, with a description of five novel 16S rRNA hemoplasmas genotypes [3]. Similarly, Sousa et al. [4] reported a high occurrence of the bacteria in ring-tailed coatis (Procyonidae: *Nasua nasua*; hereafter “coati”) in the Brazilian Pantanal, with the description of two 16S rRNA genotypes. In Paraná state, southern Brazil, hemoplasma was detected in captive coatis [5] and a putative novel, namely ‘*Candidatus* Mycoplasma haematonasua’, was evidenced through phylogeny based on two molecular markers (16S rRNA and 23S rRNA) in wild coatis [6].

Infections by hemoplasmas in domestic animals can be manifested in acute and chronic illnesses. Acute infections are characterized by anemia and acute hemolysis, anorexia, lethargy, dehydration, weight loss, and, in some cases, sudden death [7]. Chronic infection, on the other hand, can occur in apparently healthy animals, with a manifestation of clinical signs in animals that have undergone a splenectomy procedure or immunosuppression. The severity of the disease will depend on the strain or species of hemoplasma involved [7]. In wild animals, hemoplasma pathogenicity is poorly known, but infections appear to be subclinical [2]. Therefore, to understand how bacteria affect wild animals’ health, studies assessing the clinical and hematological parameters of infected animals are needed.

The genus *Bartonella* spp. (Rhizobiales: Bartonellaceae) encompasses Gram-negative, facultative intracellular α-proteobacteria that primarily infect erythrocytes and endothelial cells of a wide variety of mammals [8]. With at least 45 species described, they are considered re-emerging zoonotic agents, which can cause a plethora of clinical signs, which vary from self-limiting manifestations to fatal systemic diseases [8,9]. The transmission of *Bartonella* spp. is also associated with hematophagous arthropod vectors, such as fleas, lice, sandflies [10] and, more recently, ticks [11]. Considering a large number of mammal reservoirs and arthropod vectors, the transmission of Bartonellaceae leads to a challenging epidemiological scenario, especially in regions where wild animals are in close contact with humans and domestic animals. Regarding the occurrence of Bartonellaceae in Procyonidae mammals, *Bartonella henselae* and *Bartonella koehlerae* were detected in *P. lotor* in the state of Colorado and St. Simons Island, USA [12,13].

To the best of the authors’ knowledge, there are no reports of *Bartonella* sp. in Procyonidae mammals from Brazil, and although hemoplasmas have been reported in coatis from Brazil, there is no information about the infection on health parameters of naturally infected animals. The present study: (i) estimates the fluctuation of hemoplasmas and *Bartonella* spp. bacterial load in free-living coatis in a longitudinal study in two urban areas in central-western Brazil by quantitative PCR (qPCR), (ii) investigates the presence of hemoplasmas and *Bartonella* spp. DNA in ectoparasites collected from coatis, and (iii) assess the correlation between the two bacterial infections and coatis’ red blood cell parameters.

## 2. Material and Methods

### 2.1. Blood and Ectoparasites Sampling

Between March 2018 and January 2019, coatis were sampled every three months for 10 consecutive days in two urban areas: a conservation unit ‘Parque Estadual do Prosa’ (PEP) (−20.44987, −54.56529) and a residential ‘Vila da Base Aérea’ (VBA) (−20.47163, −54.65405), both located in Campo Grande city, Mato Grosso do Sul State, Midwestern Brazil. They were anesthetized with an association of Tiletamine hydrochloride and Zolazepam hydrochloride (Telazol, Zoetis^®^, Parsippany-Troy Hills, NJ, USA—7 mg/Kg, Intramuscularly) and were marked with numbered colored earrings and had a microchip implanted in the subcutaneous tissue between the shoulder blades [14]. Age was estimated according to Olifiers et al. [15] in three groups: adults, subadults and puppies.

In total, 97 different coatis were sampled (42 PEP and 55 VBA; 56 females and 41 males; and 70 adults and 27 subadults). Coatis were recaptured by chance, totalizing 163 blood samples in total (first capture and recaptures). Blood was sampled from the femoral vein using tubes containing EDTA (Ethylenediamine Tetraacetic Acid) and then placed into RNAse/DNAse free cryotubes and stored in an −80 °C freezer until culturing and molecular analyses. The entire body of the captured animals was inspected, and ectoparasites were collected and stored in 100% ethanol (Merck^®^, Darmstadt, Hesse, Germany) for taxonomic identification [16,17] and further molecular analyses.

Ticks collected (n = 2242) were identified as *Amblyomma* spp. larvae (n = 838), *Amblyomma sculptum* nymphs (n = 1241), and *Amblyomma dubitatum* nymphs (n = 150). Thirteen adult ticks were identified as *A. sculptum* (n = two males and n = five females) and two males and three females of *Amblyomma ovale* [14]. Lice collected (n = 59) were identified as *Neotrichodectes (Nasuicola) pallidus* (n = 27 nymphs, n = 15 females and n = 17 males) *(manuscript submitted*).

### 2.2. DNA Extraction from Coatis’ Blood, Ectoparasite Samples and Conventional PCR (cPCR) for Mammal Gadph, Tick 16S rRNA, and Insects Cox-1 Endogenous Genes

DNA was extracted from 200 μL of blood using Illustra Blood Mini Kit (GE Healthcare^®^, Chicago, IL, USA), according to the manufacturer’s instructions. In the case of ticks, while DNA from tick larvae and nymphs was extracted in pools of up to 10 and 5 individuals, respectively, collected from the same host, DNA was extracted from adults individually. Regarding lice, individual specimens collected from individual coatis were subjected to DNA extraction; when more than one developmental stage was found in individual coatis, DNA was extracted from louse pools (e.g., pools of nymphs and pools of female adults were extracted separately from an individual). DNA extraction of ticks and lice was performed using the Biopur Mini Spin Plus kit (Mobius^®^, Pinhais, Paraná, Brazil), according to the manufacturer’s instructions. DNA extracted samples were subjected to endogenous gene control cPCR assays {*gapdh* gene (Glyceraldehyde 3-phosphate dehydrogenase) for blood [18], 16S rRNA gene for ticks [19] and *cox1* gene for louse [20]}. Ultra-pure sterile water (Life Technologies^®^, Carlsbad, CA, USA) was used as a negative control in all cPCR assays.

### 2.3. Quantitative PCR Assay (qPCR) and Molecular Characterization of Hemoplasmas

In order to detect and quantify the presence of hemoplasmas DNA, positive samples in cPCR assays for the endogenous genes were subjected to a Sybr Green quantitative PCR (qPCR) assay based on the 16S rRNA gene for pan-hemoplasmas [21]. All analyses were performed according to the standards established by MIQE (Minimum Information for Publication of Quantitative real-time PCR Experiments) [22]. Each DNA sample was subjected to qPCR assay in duplicate. The amplification reactions were carried out in a CFX96 Thermal Cycler (BioRad^®^, Hercules, Coralville, CA, USA). Serial dilutions were performed to construct standard curves with different concentrations (2.0 × 10^7^ to 2.0 × 10^0^ copies/μL) of a plasmid encoding a fragment of 104 bp of the 16S rRNA gene of *Mycoplasma haemofelis* (pIDTSMART; Integrated DNA Technologies Inc., Coralville, IA, USA), diluted in Tris-EDTA (TE; 10 mmol/l, Tris-HCl, 0.1 mmol/l, EDTA) (pH 8.0). The number of plasmid copies was determined by the formula (XG/μL DNA/[Plasmid Length (BP) × 660]) × 6.022 × 10^23^ × plasmid copies/μL [22]. Ultra-pure sterile water was used as a negative control.

Due to financial limitations, it was not possible to sequence all positive samples. Therefore, 15 samples were randomly selected for sequencing amplicons obtained from two cPCR assays based on the 16S rRNA gene, using two sets of primers (fragments of ~800 bp for each protocol) [23]. The samples sequenced for 16S rRNA were also submitted to a cPCR assay based on the 23S rRNA (~900 bp) [24] gene. *Mycoplasma parvum* DNA obtained from a naturally infected swine [25] and ultra-pure sterile water were used as positive and negative controls, respectively.

### 2.4. Culturing for Bartonella *spp.*

All blood samples (200 μL) were cultured in 2 mL of *Bartonella*-Alpha Proteobacteria Growth Medium (BAPGM) and were kept under agitation at 37 °C with 5% CO_2_ for 7 days. Then, 200 μL of each enrichment liquid culture containing blood sample were seeded onto a solid culturing medium of enriched chocolate agar [26,27] and maintained under the same conditions for up to four weeks. Colonies suggestive of Bartonellaceae were individually collected, subjected to DNA extraction by the boiling method [28] and subjected to a qPCR assay for *Bartonella* spp., based on the *nuoG* gene [29]. The remainder of each enrichment liquid culture was also subjected to DNA extraction and qPCR assay. All culture protocols followed Furquim et al. [30].

### 2.5. Quantitative PCR Assay (qPCR) and Molecular Characterization of Bartonella *spp.*

The positive samples for the endogenous genes protocols were then submitted to a quantitative screening qPCR for *Bartonella* spp. based on the NADH dehydrogenase gamma subunit (*nuoG*) gene, as previously described [29]. Serial dilutions were performed to construct standard curves with different concentrations (2.0 × 10^7^ to 2.0 × 10^0^ copies/μL) of the plasmid, which encodes an 83 bp fragment of *Bartonella henselae nuoG* gene (pIDTSMART; Integrated DNA Technologies), diluted in Tris-EDTA. Ultra-pure sterile water was used as a negative control in qPCR assays. The diluted plasmids encoding a fragment of the *nuoG* gene of *B. henselae* were used as positive controls. Molecular characterization was performed with PCR assays according to Furquim et al. [29]. The number of plasmid copies was determined by the formula (XG/μL DNA/ [Plasmid Length (BP) × 660]) × 6.022 × 10^23^ × plasmid copies/μL [22]. *Bartonella henselae* DNA from a naturally infected cat was used as a positive control [31]. Ultra-pure sterile water was used as a negative control in cPCR assays.

### 2.6. Agarose gel Electrophoresis, Sequencing and Phylogenetic Analyses

Results of the cPCR were visualized in 1% agarose gel stained by ethidium bromide solution in a UV transilluminator (ChemiDoc MP Imaging System, Bio Rad^®^, Hercules, CA, USA), and sequencing of amplicons was performed by Sanger method [32] using ABI PRISM 3730 DNA Analyzer (Applied Biosystems, Waltham, MA, USA). Electropherogram results and consensus sequences were analyzed using Phred-Phrap software version 23 [33]. The BLASTn tool was used to browse and compare with sequences from the GenBank^®^ international database (https://www.ncbi.nlm.nih.gov/genbank/ accessed on 30 October 2022). All sequences obtained in the present study were deposited in GenBank^®^. For phylogenetic analyses, 23S rRNA and 16S rRNA (only large) hemoplasma sequences obtained were used. The alignment was performed using MAFFT software (https://mafft.cbrc.jp/alignment/server/ accessed on 30 October 2022), and Bayesian analyses were performed using the CIPRES gateway [34]. The phylogenetic tree edition and rooting (outgroup) were performed using TreeGraph 2.0 beta software [35]. The pairwise distance matrix among sequences of 16S rRNA gene of hemoplasmas detected in the present study was estimated using the Mega-X software version 10.1.8.

### 2.7. Hematological and Path Analyses

Red blood cells (10^6^/μL), hemoglobin (g/dL), and hematocrit (mm^3^) were determined using a hematology analyzer device (POCH-iV 100, Syxmex^®^, São Paulo, Brazil), standardized for ring-tailed coatis according to manufacturers’ instructions. Individuals of infected coatis for each hemoplasma genotype detected were expressed by the frequency of occurrence between area (PEP × VBA), sex (Female × Male), and age (Adults × Imatures—puppies and subadults). In order to test possible associations between these variables with the infections, Chi-squared tests were used.

To determine the correlation of hemoplasma bacterial load to Anemia indicators, we performed a path analysis using the data obtained from the first capture of each individual (without recaptures), according to Santos et al. [36]. Analyses were performed separately for each hemoplasma genotype. We created the Anemia indicators using red blood cells, hemoglobin, and hematocrit values through a dimensionality reduction by Principal Coordinate Analysis. We used an r-value > 0.60 to interpret the results (positive or negative effect) of the path analysis. We considered the variables statistically significant for *p*-values < 0.05 for all analyses. All data were analyzed using R software (R Development Core Team, 2015).

## 3. Results

### 3.1. DNA Extraction from Coatis’ Blood and Ectoparasites Samples and Conventional PCR (PCR) Assays for Endogenous Genes

All DNA extracted from 163 coatis’ blood samples were positive in the PCR targeting the *gapdh* gene and were then submitted to further molecular analyses. On the other hand, 248 out of 265 tick DNA pools were positive at the PCR targeting the 16S rRNA (nine *Amblyomma* sp. larvae pools, five *A. sculptum* nymphs and three *A. dubitatum* nymphs were negative, and then excluded from the other PCR assays). All 42 lice DNA samples (including pools and individual extractions) were positive in the PCR assay targeting the *cox*1 endogenous gene and were submitted to further molecular analyses.

### 3.2. Quantitative PCR Assay (qPCR) for Hemoplasmas

Regarding the first capture, 86/97 (88.6%) coatis were positive in the qPCR for hemoplasmas. When considering captures and recaptures, 146/163 (89.6%) coati blood DNA samples were positive for hemoplasmas. Based on the Tm (Melting Temperature) retrieved from 16S rRNA-based qPCR assay, two different genotypes were detected. A total of 71% (69/97) coatis were positive for a hemoplasma genotype (myc1) presenting Tm = 79–79.5 °C; 17% (17/97) individuals were positive for a hemoplasma genotype (myc2) presenting Tm = 77–77.5 °C; 12% (11/97) coatis were negative for hemoplasmas. The quantification of positive samples ranged from 2.56 × 10^0^ to 1.26 × 10^4^ copies of a 16S rRNA gene fragment/μL from #myc1 and 6.72 × 10^0^ to 1.41 × 10^3^ from myc2. All quantifications are shown in Board 1—Supplementary File. No double Tm peaks were observed when analyzing individual samples, suggesting, therefore, the absence of coinfections by myc1 and myc2 genotypes in coatis’ blood samples.

Regarding tick DNA samples, 25/248 (10%) were positive for hemoplasmas [15 *Amblyomma* spp. larvae pools (15/25—60%), two *A. dubitatum* nymph pools (2/25—8%) and eight *A. sculptum* nymph pools (8/25—32%)]. Based on the Tm analysis, the hemoplasma genotype detected in the positive ticks corresponded to myc1. It was not possible to quantify the number of copies of a fragment of the hemoplasma 16S rRNA gene fragment in 21 tick DNA samples due to the low amount of the target DNA (Monte Carlo effect). The quantification of the four positive (one *Amblyomma* sp. larvae pool, two *A. sculptum* nymph pools and two *A. dubitatum* nymph pool) samples ranged from 1.25 × 10^0^ to 2.75 × 10^0^ copies of the hemoplasma 16S rRNA gene fragment/μL (Tm = 79/79.5 °C). The 25 positive tick DNA samples were collected from positive coatis presenting a quantification ranging from 3.45 × 10^1^ to 2.02 × 10^4^ copies of the hemoplasma 16S rRNA gene fragment/μL, with Tm of 79/79.5 °C (myc1). All 42 lice DNA samples were negative in the qPCR for hemoplasmas.

From recaptured animals, two (PEP18 and VBA19) were negative in the qPCR for hemoplasmas in all recaptures. All the other animals presented fluctuations in the estimated hemoplasma bacterial load, albeit no pattern of bacteria load was observed during recaptures (Table 1). Based on the Tm analysis, only one hemoplasma genotype was found infecting coatis in different recapture times.

### 3.3. Molecular Characterization of Hemoplasmas

**Figure 1:**
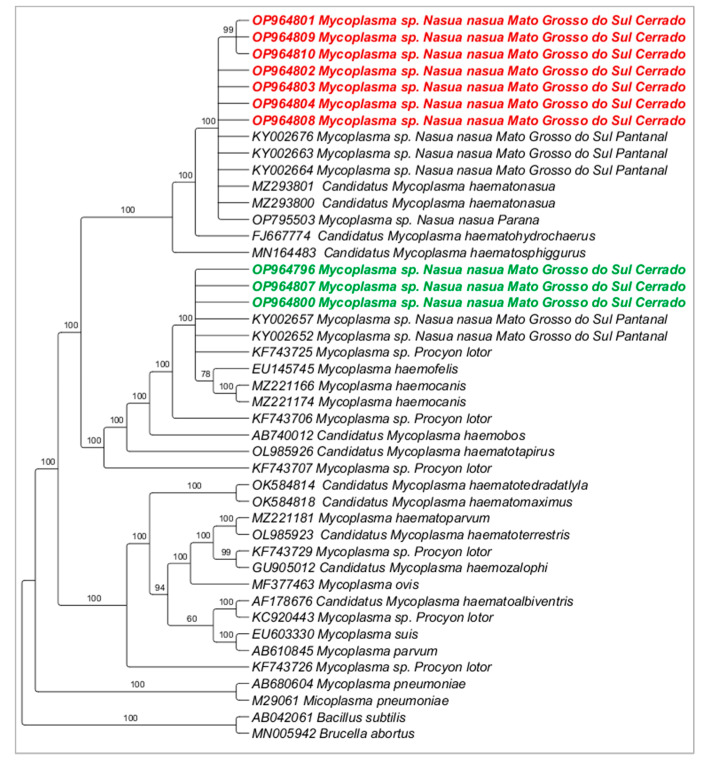
Phylogenetic tree based on Bayesian method of an alignment of 1252 bp and GTR+G evolutionary model of the 16S rRNA gene of hemoplasmas detected in coati blood samples from urban forested fragments of Campo Grande city, Mato Grosso do Sul state, Brazil. *Bacillus subtilis* (AB042061) and *Brucella abortus* (MN005942) were used as outgroups. Numbers at the nodes correspond to posterior probabilities. Sequences detected in the present study were highlighted in red bold (myc1) and in green bold (myc2).

Fifteen samples (seven from PEP and eight from VBA) were randomly chosen for sequencing of both 16S rRNA gene fragments, resulting in sequences that ranged from 646 to 1124 base pairs (bp) (GenBank^®^ Accession numbers: OP964769–OP964810). Phylogenetic analyses (Bayesian method) using only the long fragments obtained clustered the sequences detected in the present study into two clades: while clade #1 was composed of myc1 (detected in the present study), hemoplasma previously detected in coatis from Pantanal (Mato Grosso do Sul state), and ‘*Candidatus* M. haematonasua’, previously detected in coatis from Iguaçu National Park (Paraná state), clade #2 was composed by myc2 (detected in the present study) and *Mycoplasma haemofelis* and *Mycoplasma haemocanis* (Figure 1). While myc1 was represented by amplicons showing Tm = 79–79.5 °C, myc2 was represented by amplicons showing Tm = 77–77.5 °C.

**Figure 2:**
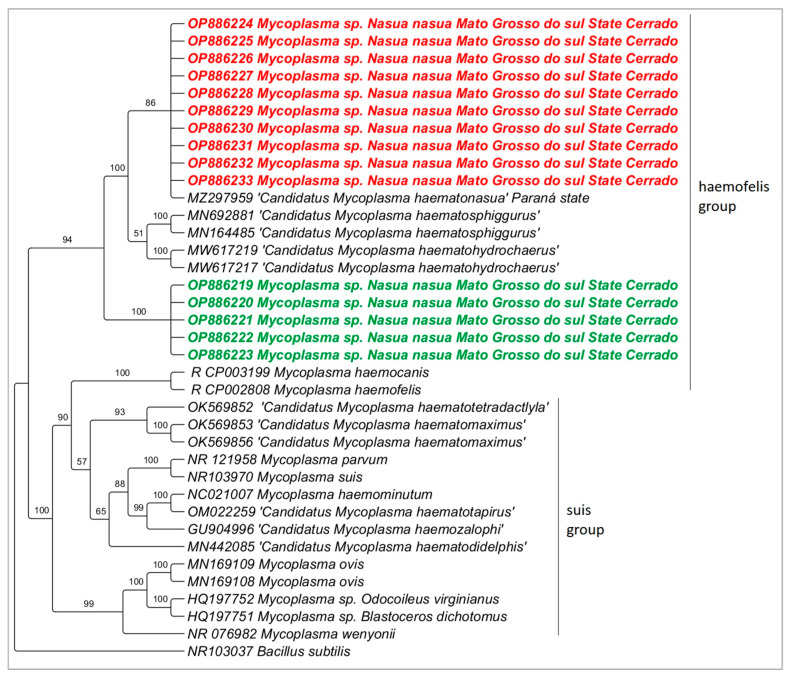
Phylogenetic tree based on Bayesian method of an alignment of 890 bp and TN+G evolutionary model of the 23S rRNA gene of hemoplasmas detected in coati blood samples from urban fragments areas of Campo Grande city, Mato Grosso do Sul state, Brazil. *Bacillus subtilis* (NR103037) was used as outgroup. Numbers at the nodes correspond to posterior probabilities. Sequences detected in the present study were highlighted in red bold (myc1) and in green bold (myc2).

Regarding the 23S rRNA gene, sequences ranging from 724 to 764 bp were obtained (GenBank^®^ Accession numbers: OP886219–OP886233). Phylogenetic analyses (Bayesian method) clustered the sequences detected in the present study into one large clade composed of two sub-clades: while one sub-clade was composed of the myc1 detected in the present study and ‘*Candidatus* Mycoplasma haematonasua’, ’*Candidatus* Mycoplasma haematosphiggurus’ and ‘*Candidatus* Mycoplasma haematohydrochaerus’, the second sub-clade was composed of myc2 detected in the present study (Figure 2).

Based on the 16S rRNA, the pairwise distance between the myc1 genotype and ‘*Candidatus* M. haematonasua’ ranged from 0 to 0.1%; genotypes myc1 and myc2 showed genetic divergence ranging from 0 to 1.3%; myc2 genotype and *Mycoplasma* sp. from *Procyon lotor* showed a genetic divergence of 0. When comparing the myc2 genotype and *M. haemofelis* and *M. haemocanis*, genetic divergence from 0.005 to 0.006 was found.

### 3.4. Culturing and qPCR Assay (qPCR) for Bartonella *spp.*

None of the 163 coatis’ blood samples were positive, neither at the qPCR assay for *Bartonella* spp. from blood DNA samples nor at liquid and solid cultures followed by qPCR. All *N. pallidus* DNA samples were negative in the qPCR for *Bartonella* spp. Four tick samples were positive in the qPCR for *Bartonella* spp.: two *Amblyomma* sp. larvae pools (one from PEP and one from VBA; Cq values of 37.2 and 38.9) and two *A. dubitatum* nymph pools (one from PEP and one from VBA; Cq values of 36.9 and 38.5). The quantification of the number of copies of a fragment of the *Bartonella nuoG* gene fragment was not possible due to the Monte Carlo effect. None tick DNA sample positive in the qPCR was positive in the conventional PCR assays for *Bartonella* sp. targeting the *gltA*, *rpoB*, *ftsZ*, *pap-31*, *ribC*, and *nuoG* molecular markers.

### 3.5. Hematological Analyses and Path Analysis

All hematological analyses are shown in Board 2—Supplementary File. Our analysis showed no association between the analyzed variables (area, sex, and age) and the positivity for myc1 and myc2. Regarding area, the occurrence rates of coatis positive to myc1 were 81% (34/42) in PEP and 64% (35/55) in VBA (Chi^2^ = 2.68, *p* = 0.10). On the other hand, 9% (04/42) were positive for myc2 in PEP and 24% (13/55) in VBA (Chi^2^ = 2.38, *p* = 0.12). The presence of DNA of myc1 was identified in 75% (42/56) of females and 66% (27/41) of males (Chi^2^ = 0.57, *p* = 0.45). On the other hand, myc2 was detected in 18% (10/56) of females and 17% (7/41) of males (Chi^2^ = 1.10, *p* = 1). Lastly, 77% (54/70) adults and 55% (15/27) immatures (puppies and subadults) were positive for myc1 (Chi^2^ = 3.43, *p* = 0.06), and 14% (10/70) and 26% (7/27) adults and subadults, respectively, were positive to myc2 (Chi^2^ = 1.11, *p* = 0.29). Furthermore, there was also no association between hemoplasma bacterial load (CMH: *p* = 0. 89, CMP: *p* = 0.81) and the anemia indicators for any of the hemoplasma genotype (myc1 *p* = 0. 89; myc2 *p* = 0.67).

## 4. Discussion

Herein, the high rates of coatis positive for hemoplasmas corroborate previous studies performed in Brazil. For instance, Sousa et al. [4] found 77.4% coatis positive for hemoplasmas in the Brazilian Pantanal, with two different genotypes: one closely related to the capybara (*Hydrochoerus hydrochaeris*)-associated hemoplasma in Brazil, and another one closely related to *Mycoplasma haemofelis*. In the present study, a similar result was observed, with the formation of two different clades with two distinct detected sequences. Interestingly, the melting temperature (Tm) allowed the differentiation between the two hemoplasma genotypes detected in the present study. Therefore, the pan-hemoplasma qPCR based on the 16S rRNA can be a good tool to screen coatis’ blood and ectoparasite samples in areas where more than one hemoplasma genotype circulate in coatis.

Even though the main route of transmission of hemoplasmas is still unknown, alternative routes of transmission have been demonstrated, such as vertical, blood transfusion, and aggressive interactions among cats and wild rodents [37,38,39,40,41]. Sousa et al. [4] pointed out that other routes of transmission, such as agonistic encounters between individuals from different groups and inside the same group, might have more importance in the transmission of hemoplasmas among these animals, mainly due to the gregarious behavior of this mammal species, with the formation of large familiar groups [42]. The positivity for hemoplasmas among immature coatis (55%) might also suggest the possibility of vertical transmission of hemoplasmas among coatis, which should be further investigated in the future.

Regarding recaptured animals, we observed a fluctuation in the estimated hemoplasma bacterial load among the successful recaptures, although we could not observe a pattern of such fluctuation of quantification. While some animals presented constant hemoplasma bacterial load throughout the recaptures, others showed an increase or decrease in bacterial load. The lack of pattern on the estimated quantification of hemoplasmas might be due to the non-standardization among samplings dates since all recaptures were performed by chance. Based on Tm analysis, all recaptured animals were infected with the same hemoplasma genotypes, and no co-infection of myc1 and myc2 was observed.

In the present study, no association was found between hemoplasma bacterial load (estimate quantification of a fragment of the 16S rRNA gene of hemoplasmas by qPCR) and red blood cell parameters. According to the results found herein, the infection by myc1 and myc2 seems not to cause anemia in wild coatis. The impact of these infections on coatis kept in captivity and under stressful conditions should be investigated in the future. It is known that some hemoplasmas species that infect domestic animals (e.g., *Mycoplasma suis*, *Mycoplasma haemocanis*, *Mycoplasma haemofelis*) can cause moderate to severe disease in their hosts [4]. Several studies have indicated that hemoplasma infection can lead to a decrease in red blood cell parameters [4,43,44,45], which can be associated with bacterial load [46]. On the other hand, some studies did not find such an association, which can be due to the involved or the presence of chronic carriers, with no red blood cell abnormalities among the tested animals [2,47].

Despite the efforts of a multi-approach diagnostic workflow, *Bartonella* sp. was not detected in coati blood samples. Interestingly, *Bartonella* spp. DNA was detected by qPCR in 10% of ticks collected from negative coatis. The tick samples positive in the qPCR assay for *Bartonella* spp. were negative in cPCR based on six molecular markers, likely because of the low amount of the targeted DNA, which might be below the limit of the detection of the conventional PCR assays used for molecular characterization. Transmission of *Bartonella* species occurs mainly through fleas and lice [48,49]. The positivity of *A. dubitatum* nymphs found in the present study might be associated with the feeding of a Bartonellaceae-infected host in the larval stage. Interestingly, *Bartonella* DNA was detected in larvae pools collected from negative coatis. Due to the low quantity of DNA copies in ticks, the detection of *Bartonella* DNA in nymphs and larvae might merely indicate the presence of remnant blood with *Bartonella* sp. DNA from a previous blood meal, without any involvement of this tick species in *Bartonella* sp. transmission. The role of *Amblyomma* ticks in the transmission and maintenance of *Bartonella* spp. should be further investigated by experimental studies.

## 5. Conclusions

Hemoplasmas are endemic in coatis from urban areas in Midwestern Brazil, with the presence of two hemoplasma genotypes. The infection of wild coatis by the two hemoplasma genotypes was not associated with anemia. *Amblyomma* spp. ticks seem not to play an important role in the transmission of hemoplasmas among coatis in the two studied areas. *Bartonella* spp. seems not to occur in wild coatis parasitized by *Amblyomma* ticks and *N. pallidulus* louse in forested urban areas in midwestern Brazil.

## Figures and Tables

**Table 1:** Identification of coatis (*Nasua nasua*) sampled between 2018–2019 in two peri-urban areas from Campo Grande do Sul, Mato Grosso do Sul, Brazil, with identification of the animal (ID), sex, qPCR melting temperature (Tm), detected hemoplasma genotype (myc1 with Tm = 79/79.5 °C or myc2 with Tm = 77–77.5 °C), and quantification of the detected hemoplasma genotype based on qPCR assay based on the 16S rRNA gene during successive recaptures.

ID	Sex	1C	2C	3C	4C	5C	6C
PEP01	M	2.02 × 10^4^ (Tm = 79.5) myc1	2.66 × 10^2^ (Tm = 79.5) myc1	1.18 × 10^3^ (Tm = 79.5) myc1	x	x	x
PEP02	M	6.97 × 10^2^ (Tm = 79.5) myc1	8.76 × 10^2^ (Tm = 79.5) myc1	x	x	x	x
PEP04	M	Negative	6.83 × 10^2^ (Tm = 79.5) myc1	3.80 × 10^3^ (Tm = 79.5) myc1	x	x	x
PEP05	M	5.28 × 10^1^ (Tm = 77.5) myc2	3.43 × 10^2^ (Tm = 77.5) myc2	x	x	x	x
PEP12	F	1.65 × 10^2^ (Tm = 79/79.5) myc1	4.09 × 10^2^ (Tm = 79/79.5) myc1	x	x	x	x
PEP17	F	2.86 × 10^1^ (Tm = 79/79.5) myc1	1.83 × 10^2^ (Tm = 79/79.5) myc1	x	x	x	x
PEP18	F	Negative	Negative	x	x	x	x
PEP20	F	1.97 × 10^2^ (Tm = 79/79.5) myc1	3.63 × 10^2^ (Tm = 79/79.5) myc1	x	x	x	x
PEP23	F	1.89 × 10^2^ (Tm = 79.5) myc1	1.36 × 10^3^ (Tm = 79.5) myc1	x	x	x	x
PEP24	F	1.12 × 10^2^ (Tm = 79/79.5) myc1	1.65 × 10^2^ (Tm = 79/79.5) myc1	x	x	x	x
PEP31	M	1.38 × 10^3^ (Tm = 79.5) myc1	4.66 × 10^3^ (Tm = 79.5) myc1	x	x	x	x
PEP32	M	1.61 × 10^3^ (Tm = 79.5) myc1	1.04 × 10^3^ (Tm = 79.5) myc1	x	x	x	x
PEP43	M	6.30 × 10^2^ (Tm = 79.5) myc1	1.58 × 10^3^ (Tm = 79.5) myc1	x	x	x	x
VBA01	M	2.32 × 10^2^ (Tm = 79/79.5) myc1	1.36 × 10^1^ (Tm = 79/79.5) myc1	x	x	x	x
VBA03	F	5.02 × 10^1^ (Tm = 79.5) myc1	3.95 × 10^2^ (Tm = 79.5) myc1	2.12 × 10^2^ (Tm = 79.5) myc1	3.49 × 10^2^ (Tm = 79.5) myc1	1.55 × 10^2^ (Tm = 79.5) myc1	5.39 × 10^1^ (Tm = 79.5) myc1
VBA05	M	4.00 × 10^2^ (Tm = 79.5) myc1	1.70 × 10^2^ (Tm = 79.5) myc1	x	x	x	x
VBA06	M	1.62 × 10^2^ (Tm = 79.5) myc1	5.74 × 10^2^ (Tm = 79.5) myc1	x	x	x	x
VBA07	M	3.18 × 10^2^ (Tm = 79.5) myc1	3.52 × 10^2^ (Tm = 79.5) myc1	2.90 × 10^2^ (Tm = 79.5) myc1	2.96 × 10^2^ (Tm = 79.5) myc1	x	x
VBA08	F	2.25 × 10^2^ (Tm = 79.5) myc1	5.49 × 10^1^ (Tm = 79.5) myc1	8.98 × 10^2^ (Tm = 79.5) myc1	x	x	x
VBA09	M	Negative	Negative	Negative	Negative	x	x
VBA10	F	1.05 × 10^3^ (Tm = 77.5) myc2	6.79 × 10^1^ (Tm = 77.5) myc2	7.16 × 10^1^ (Tm = 77.5) myc2	x	x	x
VBA11	M	Negative	1.88 × 10^4^ (Tm = 79/79.5) myc1	7.11 × 10^3^ (Tm = 79/79.5) myc1	x	x	x
VBA12	F	9.60 × 10^0^ (Tm = 79) myc1	7.47 × 10^0^ (Tm = 79) myc1	x	x	x	x
VBA16	F	5.16 × 10^2^ (Tm = 77.5) myc2	1.31 × 10^1^ (Tm = 77.5) myc2	2.62 × 10^1^ (Tm = 77.5) myc2	1.10 × 10^2^ (Tm = 77.5) myc2	x	x
VBA17	F	8.10 × 10^3^ (Tm = 79.5) myc1	9.79 × 10^1^ (Tm = 79.5) myc1	x	x	x	x
VBA19	F	2.51 × 10^2^ (Tm = 79.5) myc1	5.48 × 10^1^ (Tm = 79.5) myc1	x	x	x	x
VBA21	M	3.59 × 10^0^ (Tm = 77/77.5) myc2	5.64 × 10^0^ (Tm = 77/77.5) myc2	3.00 × 10^2^ (Tm = 77/77.5) myc2	4.94 × 10^0^ (Tm = 77/77.5) myc2	2.85 × 10^0^ (Tm = 77/77.5) myc2	5.98 × 10^4^ (Tm = 77/77.5) myc2
VBA22	F	5.14 × 10^0^ (Tm = 79) myc1	5.39 × 10^0^ (Tm = 79) myc1	x	x	x	x
VBA23	F	7.45 × 10^0^ (Tm = 77.5) myc2	7.45 × 10^0^ (Tm = 77.5) myc2	x	x	x	x
VBA25	M	Negative	Negative	1.70 × 10^1^ (Tm = 79) myc1	3.33 × 10^0^ (Tm = 79) myc1	9.03 × 10^3^ (Tm = 79.5) myc1	x
VBA29	M	2.18 × 10^2^ (Tm = 79/79.5) myc1	4.65 × 10^2^ (Tm = 79/79.5) myc1	x	x	x	x
VBA38	F	6.33 × 10^0^ (Tm = 79) myc1	3.18 × 10^0^ (Tm = 79) myc1	x	x	x	x
VBA41	F	2.05 × 10^3^ (Tm = 79/79.5) myc1	2.33 × 10^2^ (Tm = 79/79.5) myc1	x	x	x	x
VBA44	M	2.09 × 10^2^ (Tm = 77.5) myc1	2.39 × 10^2^ (Tm = 77.5) myc1	1.88 × 10^2^ (Tm = 77.5) myc1	x	x	x
VBA55	M	2.03 × 10^2^ (Tm = 79) myc1	2.09 × 10^2^ (Tm = 79) myc1	x	x	x	x

PEP: Parque Estadual do Prosa; VBA: Vila da Base Aérea; M: Male; F: Female; 1C: First capture; 2C: Second capture; 3C: Third capture; 4C: Fourth capture; 5C: Fifth capture; 6C: Sixth capture; x: non-captured.

## Data Availability

The datasets generated and analyzed during the current study are available in the NCBI GenBank^®^ Nucleotide platform and can be accessed through accession numbers: OP964769–OP964810 for 16SrRNA gene and OP886219–OP886233 for 23SrRNA gene.

## Supplementary Materials

The following supporting information can be downloaded at: https://www.mdpi.com/article/10.3390/pathogens12040538/s1, Table S1. Identification of coatis sampled and recaptured in the two locations (PEP and VBA), field number, sex, age group and qPCR positivity for hemoplasmas based on the 16S rRNA gene. The quantification cycle average values (Cq), quantification average (number of copies of a 16S rRNA gene fragment per µL), melting temperature (Tm), and positivity in conventional PCR assays for two different regions of the 16S RNA gene, named here as “first” (900 bp) and “second” (900 pb) fragments, and samples selected for sequencing an 800 bp fragment of the 23S rRNA gene.

## References

[1] Di Cataldo S., Hidalgo-Hermoso E., Sacristán I., Cevidanes A., Napolitano C., Hernández C.V., Cabello J., Muller A., Millán J. (2020). Hemoplasmas are endemic and cause asymptomatic infection in the endangered Darwin’s fox (*Lycalopex fulvipes*). Appl. Environ. Microbiol..

[2] Millán J., Di Cataldo S., Volokhov D.V., Becker D.J. (2021). Worldwide occurrence of haemoplasmas in wildlife: Insights into the patterns of infection, transmission, pathology and zoonotic potential. Transbound. Emerg. Dis..

[3] Volokhov D.V., Hwang J., Chizhikov V.E., Danaceau H., Gottdenker N.L. (2017). Prevalence, Genotype Richness, and Coinfection Patterns of Hemotropic Mycoplasmas in Raccoons (*Procyon lotor*) on Environmentally Protected and Urbanized Barrier Islands. Appli. Environ. Microbiol..

[4] Sousa K.C.M., Herrera H.M., Secato C.T., Oliveira A.D.V., Santos F.M., Rocha F.L., Barreto W.T.G., Macedo G.C., de Andrade Pinto P.C.E., Machado R.Z. (2017). Occurrence and molecular characterization of hemoplasmas in domestic dogs and wild mammals in a Brazilian wetland. Acta Trop..

[5] Cubilla M.P., Santos L.C., De Moraes W., Cubas Z.S., Leutenegger C.M., Estrada M., Lindsay L.L., Trindade E.S., Franco C.R.C., Vieira R.F.C. (2017). Microscopic and molecular identification of hemotropic mycoplasmas in South American coatis (*Nasua nasua*). Comp. Immunol. Microbiol. Infect. Dis..

[6] Collere F.C.M., Delai R.M., Ferrari L.D.R., da Silva L.H., Fogaça P.L., Rodrigues A.N., Gonçalves D.D., Baggio R.A., Moraes M.F.D., Hoppe E.G.L. (2021). ‘*Candidatus* Mycoplasma haematonasua’ and tick-borne pathogens in ring-tailed coatis (*Nasua nasua*, Linnaeus, 1976) from the Iguaçu National Park, Paraná State, southern Brazil. Transb. Emerg. Dis..

[7] Messick J.B. (2004). Hemotrophic mycoplasmas (hemoplasmas): A review and new insights into pathogenic potential. Vet. Clin. Pathol..

[8] Breitschwerdt E.B., Maggi R.G., Chomel B.B., Lappin M.R. (2010). Bartonellosis: An emerging infectious disease of zoonotic importance to animals and human beings. J. Vet. Emerg. Crit. Care.

[9] Okaro U., Addisu A., Casanas B., Anderson B. (2017). *Bartonella* species, an emerging cause of blood-culture-negative endocarditis. Clin. Microbial. Rev..

[10] Billeter S.A., Levy M.G., Chomel B.B., Breitschwerdt E.B. (2008). Vector transmission of *Bartonella* species with emphasis on the potential for tick transmission. Med. Vet. Entomol..

[11] Wechtaisong W., Bonnet S.I., Lien Y.Y., Chuang S.T., Tsai Y.L. (2020). Transmission of *Bartonella henselae* within *Rhipicephalus sanguineus*: Data on the Potential Vector Role of the Tick. PLoS Negl. Trop. Dis..

[12] Hwang J., Gottdenker N.L. (2013). *Bartonella* species in raccoons and feral cats, Georgia, USA. Emerg. Infect. Dis..

[13] Bai Y., Gilbert A., Fox K., Osikowicz L., Kosoy M. (2016). *Bartonella rochalimae* and *B. vinsonii* subsp. berkhoffii in wild carnivores from colorado, USA. J. Wildl. Dis..

[14] Perles L., Martins T.F., Barreto W.T.G., Carvalho de Macedo G., Herrera H.M., Mathias L.A., Labruna M.B., Barros-Battesti D.M., Machado R.Z., André M.R. (2022). Diversity and Seasonal Dynamics of Ticks on Ring-Tailed Coatis *Nasua nasua* (Carnivora: Procyonidae) in Two Urban Areas from Midwestern Brazil. Animals.

[15] Olifiers N., de Cassia Bianchi R., D’Andrea P.S., Mourao G., Gompper M.E. (2010). Estimating age of carnivores from the Pantanal region of Brazil. Wildl. Biol..

[16] Martins T.F., Onofrio V.C., Barros-Battesti D.M., Labruna M.B. (2010). Nymphs of the genus *Amblyomma* (Acari: Ixodidae) of Brazil: Descriptions, redescriptions, and identification key. Ticks Tick-borne Dis..

[17] Dantas-Torres F., Martins T.F., Muñoz-Leal S., Onofrio V.C., Barros-Battesti D.M. (2019). Ticks (Ixodida: Argasidae, Ixodidae) of Brazil: Updated species checklist and taxonomic keys. Ticks Tick-Borne Dis..

[18] Birkenheuer A., Whittington J., Neel J., Large E., Barge A., Levy M., Breitschwerdt E. (2006). Molecular characterization of a Babesia species identified in a North American raccoon. J. Wild. Dis..

[19] Black W.C., Piesman J. (1994). Phylogeny of hard- and soft-tick taxa (Acari: Ixodida) based on mitochondrial 16S rDNA sequences. PNAS.

[20] Folmer O., Black M., Hoeh W., Lutz R., Vrijenhoek R. (1994). DNA primers for amplification of mitochondrial cytochrome c oxidase subunit I from diverse metazoan invertebrates. Mol. Biol. Biotech..

[21] Willi B., Meli M.L., Lüthy R., Honegger H., Wengi N., Hoelzle L.E., Hofmann-Lehmann R. (2009). Development and application of a universal hemoplasma screening assay based on the SYBR Green PCR principle. J. Clin. Microbiol..

[22] Bustin S.A., Benes V., Garson J.A., Hellemans J., Huggett J., Kubista M., Wittwer C.T. (2009). The MIQE Guidelines: Minimum Information for Publication of Quantitative Real-Time PCR Experiments. Clin. Chem..

[23] Maggi R.G., Compton S.M., Trull C.L., Mascarelli P.E., Mozayeni B.R., Breitschwerdt E.B. (2013). Infection with hemotropic *Mycoplasma* species in patients with or without extensive arthropod or animal contact. J. Clin. Microbiol..

[24] Mongruel A.C., Spanhol B., Valente V.C., Porto J.D.M., Ogawa P.P., Otomura L., Vieira R.F.C. (2020). Survey of vector-borne and nematode parasites involved in the etiology of anemic syndrome in sheep from Southern Brazil. Braz. J. Vet. Parasitol..

[25] Sonalio K., Perles L., Gatto I.R.H., do Amaral R.B., Almeida H.M., Galdeano J.V.B., André M.R., de Oliveira L.G. (2021). Genetic diversity of emerging hemotropic mycoplasmas in domestic pigs from Brazil. Transb. Emerg. Dis..

[26] Maggi R.G., Duncan A.W., Breitschwerdt E.B. (2005). Novel chemically modified liquid medium that will support the growth of seven *Bartonella* species. J. Clin. Microbiol..

[27] Duncan A.W., Maggi R.G., Breitschwerdt E.B. (2007). A combined approach for the enhanced detection and isolation of *Bartonella* species in dog blood samples: Pre-enrichment liquid culture followed by PCR and subculture onto agar plates. J. Microbiol. Meth..

[28] Keim P., Price L.B., Klevytska A.M., Smith K.L., Schupp J.M., Okinaka R., Hugh-Jones M.E. (2000). Multiple-locus variable-number tandem repeat analysis reveals genetic relationships within *Bacillus anthracis*. J. Bacteriol..

[29] André M.R., Dumler J.S., Herrera H.M., Gonçalves L.R., de Sousa K.C., Scorpio D.G. (2015). Assessment of a quantitative 5′ nuclease real-time polymerase chain reaction using the nicotinamide adenine dinucleotide dehydrogenase gamma subunit (*nuoG*) for *Bartonella* species in domiciled and stray cats in Brazil. J. Feline. Med. Surg..

[30] Furquim M.E.C., do Amaral R., Dias C.M., Gonçalves L.R., Perles L., de Paula Lima C.A., André M.R. (2021). Genetic diversity and Multilocus Sequence Typing Analysis of *Bartonella henselae* in domestic cats from Southeastern Brazil. Acta Trop..

[31] Dias C.M., do Amaral R.B., Perles L., dos Santos Muniz A.L., Rocha T.F.G., Machado R.Z., André M.R. (2022). Multi-locus Sequencing Typing of *Bartonella henselae* isolates reveals coinfection with different variants in domestic cats from Midwestern Brazil. Acta Trop..

[32] Sanger F., Nicklen S., Coulson A.R. (1977). DNA sequencing with chain-terminating inhibitors. PNAS.

[33] Ewing B., Hillier L., Wendl M.C., Green P. (1998). Base-calling of automated sequencer traces usingPhred. I. Accuracy assessment. Genome Res..

[34] Ronquist F., Huelsenbeck J.P. (2003). MrBayes 3: Bayesian phylogenetic inference under mixed models. Bioinformatics.

[35] Stover B.C., Muller K.F. (2010). TreeGraph 2: Combining and visualizing evidence from different phylogenetic analyses. BMC Bioinform..

[36] Santos F.M., Macedo G.C., Barreto W.T.G., Oliveira-Santos L.G.R., Garcia C.M., Miranda Mourão G.D., Miraglia Herrera H. (2018). Outcomes of *Trypanosoma cruzi* and *Trypanosoma evansi* infections on health of Southern coati (*Nasua nasua*), crab-eating fox (*Cerdocyon thous*), and ocelot (*Leopardus pardalis*) in the Brazilian Pantanal. PLoS ONE.

[37] Dean R.S., Helps C.R., Jones T.J.G., Tasker S. (2008). Use of the real-time PCR to detect *Mycoplasma haemofelis* and ‘*Candidatus* Mycoplasma haemominutum’ in the saliva and salivary glands of hemoplasma infected cats. J. Feline Med. Surg..

[38] Cohen C., Shemesh M., Garrido M., Messika I., Einav M., Khokhlova I., Hawlena H. (2018). Haemoplasmas in wild rodents: Routes of transmission and infection dynamics. Mol. Ecol..

[39] Lashnits E., Grant S., Thomas B., Qurollo B., Breitschwerdt E.B. (2019). Evidence for vertical transmission of *Mycoplasma haemocanis*, but not *Ehrlichia ewingii*, in a dog. J. Vet. Int. Med..

[40] Stadler J., Willi S., Ritzmann M., Eddicks M., Hoelzle K., Hoelzle L.E. (2019). Detection of *Mycoplasma suis* in pre-suckling piglets indicates a vertical transmission. BCM Vet. Res..

[41] Kim J., Lee D., Yoon E., Bae H., Chun D., Kang J.G., Yu D.H. (2020). Clinical Case of a Transfusion-Associated Canine *Mycoplasma haemocanis* Infection in the Republic of Korea: A Case Report. Korean J. Parasitol..

[42] Barreto W.T.G., Herrera H.M., de Macedo G.C., Rucco A.C., de Assis W.O., Oliveira-Santos L.G., Porfirio G.E.M.O. (2021). Density and survivorship of the South American coati (*Nasua nasua*) in urban areas in Central–Western Brazil. Hystrix Italian J. Mammol..

[43] Harrus S., Klement E., Aroch I., Stein T., Bark H., Lavy E., Mazaki-Tovi M., Baneth G. (2002). Retrospective study of 46 cases of feline haemobartonellosis in Israel and their relationships with FeLV and FIV infections. Vet. Rec..

[44] Tasker S., Binns S.H., Day M.J., Gruffydd-Jones T.J., Harbour D.A., Helps C.R. (2003). Use of a PCR assay to assess the prevalence and risk factors for *Mycoplasma haemofelis* and ‘*Candidatus* Mycoplasma haemominutum’ in cats in the United Kingdom. Vet. Rec..

[45] Suzuki J., Sasaoka F., Fujihara M., Watanabe Y., Tasaki T., Oda S., Harasawa R. (2011). Molecular identification of ‘*Candidatus* Mycoplasma haemovis’ in sheep with hemolytic anemia. J. Vet. Med. Sci..

[46] Tasker S., Braddock J.A., Baral R., Helps C.R., Day M.J., Gruffydd-Jones T.J., Malik R. (2004). Diagnosis of feline haemoplasma infection in Australian cats using a real-time PCR assay. J. Feline Med. Surg..

[47] Willi B., Boretti F.S., Baumgartner C., Tasker S., Wenger B., Cattori V. (2006). Prevalence, risk factor analysis, and follow-up of infections caused by three feline hemoplasma species in cats in Switzerland. J. Clin. Microbiol..

[48] Bai Y., Kosoy M., Recuenco S., Alvarez D., Moran D., Turmelle A., Ellison J., Garcia D.L., Estevez A., Lindblade K. (2011). *Bartonella* spp. in Bats, Guatemala. Emerg. Infect. Dis..

[49] Morse S.F., Olival K.J., Kosoy M., Billeter S., Patterson B.D., Dick C.W., Dittmar K. (2012). Global distribution and genetic diversity of *Bartonella* in bat flies (Hippoboscoidea, Streblidae, Nycteribiidae). Infec. Genet. Evol..

