# Supplementary material for: Molecular Survey of Hemotropic Mycoplasma spp. and Bartonella spp. in Coatis (Nasua nasua) from Central-Western Brazil
Source: Pathogens. 2023 Mar 30;12(4):538. doi: 10.3390/pathogens12040538 (PMC10145911; doi:10.3390/pathogens12040538)
Supplement: Supplementary file 1 [file pathogens-12-00538-s001.zip › pathogens-2237683-supplementary.pdf]

Molecular survey of hemotropic *Mycoplasma* spp. and *Bartonella* spp. in coatis (*Nasua nasua*) from central-western Brazil, with evidence of a putative novel hemoplasma

Lívia Perles[1]; Wanessa Teixeira Gomes Barreto[2]; Filipe Martins Santos[3], Leidiane Lima Duarte[1], Gabriel Carvalho de Macedo[3]; Heitor Herrera Miraglia[2,3]; Rosangela Zacarias Machado[1]; Marcos Rogério André[1*]

[1] Vector-Borne Bioagents Laboratory (VBBL), Department of Pathology, Reproduction and One Health, School of Agricultural and Veterinarian Sciences, São Paulo State University (Unesp), Via de Acesso Prof. Paulo Donato Castellane, s/n, Zona Rural, Jaboticabal 14884-900, SP, Brazil

[2] Post-Graduation of Ecology and Conservation, Mato Grosso do Sul Federal University, Campo Grande 13471-410, MS, Brazil

[3] Laboratory of Parasitic Biology, Environmental Sciences and Farming Sustainability, Dom Bosco Catholic University, Campo Grande 13471-410, MS, Brazil

***Corresponding author:**

Prof. Dr. Marcos Rogério André, Vector-Borne Bioagents Laboratory (VBBL), Departamento de Patologia, Reprodução e Saúde Única, Faculdade de Ciências Agrárias e Veterinárias Júlio de Mesquita Filho (UNESP), Campus de Jaboticabal, Via de Acesso Prof. Paulo Donato Castellane, s/n, Zona Rural, CEP: 14884-900, Jaboticabal, São Paulo, Brazil. Phone: +55 (16) 3209-7302 Fax: +55 (16) 3202-4275. e-mail: mr.andre@unesp.br

Table S1. Identification of coatis sampled and recaptured in the two locations (PEP and VBA), field number, sex, age group and qPCR positivity for hemoplasmas based on the 16S rRNA gene. The quantification cycle average values (Cq), quantification average (number of copies of a 16S rRNA gene fragment per µL), melting temperature (Tm), positivity in conventional PCR assays for two different regions of the 16S RNA gene, named here as “first” (900pb) and “second” (900pb) fragments, and samples selected for sequencing an 800 bp fragment of the 23S rRNA gene.

Collection date	Identification number	Localization	Sex	Age	Mean Cq	Mean quantification of copies of a fragment of the16S rRNA/µL	Melting temperature	1° fragment 16S rRNA (900 bp) (Maggi et al. 2013)	2° fragment 16S rRNA (900 bp) (Maggi et al. 2013)	23S rRNA (800 bp) (Mongruel et al. 2020)
13/03/2018	PEP 02	PEP[1]	M	Adult	21,55	6,97x10^{2}	79,5	+	+	x
14/03/2018	PEP 04	PEP	M	Adult	Negative	Negative	-	-	-	x
15/03/2018	PEP 06	PEP	F	Adult	22,48	3,84x10^{2}	79	Sequenced	Sequenced	Sequenced
15/03/2018	PEP 08	PEP	F	Adult	23,47	1,62x10^{2}	79,5	+	+	x
16/03/2018	PEP 10	PEP	M	Adult	27,46	1,57x10^{1}	79,5	+	+	x
16/03/2018	PEP 11	PEP	F	Adult	22,85	3,03x10^{2}	79	+	+	x
20/03/2018	PEP 13	PEP	M	Subadult	18,41	5,27x10^{3}	79,5	+	+	x
21/03/2018	PEP 14	PEP	M	Adult	26,24	3,45x10^{1}	79	+	+	x
21/03/2018	PEP 15	PEP	F	Adult	25,92	4,21x10^{1}	79/79,5	+	+	x
22/03/2018	PEP 17	PEP	F	Adult	26,53	2,86 x10^{1}	79,5	+	+	x
30/04/2018	VBA 01	VBA[2]	M	Adult	23,27	2,32x10^{2}	79,5	+	+	x
02/05/2018	VBA 03	VBA	F	Adult	21,75	5,02 x10^{1}	79,5	+	+	x
02/05/2018	VBA 04	VBA	F	Adult	25,45	5,75x10^{1}	79,5	+	+	x
02/05/2018	VBA 05	VBA	M	Adult	22,09	4,00x10^{2}	79,5	+	+	x
02/05/2018	VBA 06	VBA	M	Adult	23,47	1,62x10^{2}	79	+	+	x
03/05/2018	VBA 07	VBA	M	Adult	22,44	3,18x10^{2}	79,5	+	+	x
04/05/2018	VBA 08	VBA	F	Adult	23,32	2,25x10^{2}	79,5	+	+	x
04/05/2018	VBA 09	VBA	M	Subadult	Negative	Negative	-	-	-	x
04/05/2018	VBA 10	VBA	F	Adult	20,92	1,05x10^{3}	77,5	+	+	x
07/05/2018	VBA 11	VBA	M	Adult	Negative	Negative	-	-	-	x
09/05/2018	VBA 12	VBA	F	Adult	28,23	9,60x10^{0}	79	-	+	x
10/05/2018	VBA 13	VBA	M	Adult	21,32	8,10x10^{2}	79,5	+	+	x
28/05/2018	PEP 18	PEP	F	Puppy	Negative	Negative	-	-	-	x
28/05/2018	PEP 19	PEP	F	Puppy	25,57	5,36 x10^{1}	77,5	+	-	x

29/05/2018	PEP 20	PEP	F	Adult	23,52	1,97x10^{2}	79,5	+	+	x
29/05/2018	PEP 21	PEP	F	Adult	25,71	4,82 x10^{1}	79,5	+	+	x
29/05/2018	PEP 22	PEP	F	Adult	23,6	1,87x10^{2}	79,5	+	+	x
30/05/2018	PEP 02B	PEP	M	Adult	21,2	8,76x10^{2}	79,5	+	+	x
30/05/2018	PEP 01B	PEP	M	Adult	16,12	2,02x10^{4}	79,5	+	+	x
30/05/2018	PEP 03B	PEP	F	Adult	22,67	3,41x10^{2}	79,5	+	+	x
30/05/2018	PEP 05B	PEP	M	Adult	25,17	5,28x10^{1}	77,5	Sequenced	Sequenced	Sequenced
30/05/2018	PEP 23	PEP	F	Adult	23,59	1,89x10^{2}	79,5	Sequenced	Sequenced	Sequenced
11/06/2018	PEP 12B	PEP	F	Adult	23,44	1,65x10^{2}	79	+	+	x
12/06/2018	PEP 24	PEP	F	Adult	24,4	1,12x10^{2}	79	+	+	x
12/06/2018	PEP 25	PEP	M	Adult	17,24	1,11x10^{4}	79,5	+	+	x
15/06/2018	PEP 04B	PEP	M	Adult	21,59	6,83x10^{2}	79,5	+	+	x
15/06/2018	PEP 17B	PEP	F	Adult	23,64	1,83x10^{2}	79	+	+	x
18/06/2018	VBA 03B	VBA	F	Adult	22,26	3,95x10^{2}	79,5	Sequenced	Sequenced	Sequenced
19/06/2016	VBA 15	VBA	F	Adult	18,41	5,27 x10^{3}	79,5	+	+	x
19/06/2016	VBA 12B	VBA	F	Adult	28,63	7,47x10^{0}	79	-	+	x
19/06/2016	VBA 08B	VBA	F	Adult	25,11	5,49 x10^{1}	79,5	+	+	x
20/06/2018	VBA 10B	VBA	F	Adult	24,79	6,79 x10^{1}	77,5	+	+	x
20/06/2018	VBA 16	VBA	F	Adult	21,7	5,16x10^{2}	77,5	+	+	x
20/06/2018	VBA 17	VBA	F	Adult	17,51	8,10 x10^{3}	79,5	+	+	x
20/06/2018	VBA 11B	VBA	M	Adult	16,23	1,88x10^{4}	79	Sequenced	Sequenced	Sequenced
21/06/2018	VBA 18	VBA	F	Adult	20,3	1,30 x10^{3}	77,5	+	+	x
21/06/2018	VBA 19	VBA	F	Adult	22,8	2,51x10^{2}	79,5	+	+	x
22/06/2018	VBA 20	VBA	M	Adult	25,08	5,61 x10^{1}	79,5	+	+	x
25/06/2018	VBA 21	VBA	M	Puppy	29,26	3,59x10^{0}	77	-	-	x
25/06/2018	VBA 07B	VBA	M	Adult	22,29	3,52x10^{2}	79,5	+	+	x
26/06/2018	VBA 22	VBA	F	Puppy	28,71	5,14x10^{0}	79	-	-	x
26/06/2018	VBA 23	VBA	F	Adult	28,16	7,45x10^{0}	77,5	+	-	x
26/06/2018	VBA 24	VBA	M	Subadult	28,56	5,71x10^{0}	79	-	-	x

26/06/2018	VBA 09B	VBA	M	Subadult	Negative	Negative	-	-	-	x
26/06/2018	VBA 25	VBA	M	Puppy	Negative	Negative	-	-	-	x
29/06/2018	VBA 26	VBA	F	Adult	28,61	$5{,}51x10^0$	77	-	-	x
30/07/2018	PEP 23B	PEP	F	Adult	20,24	$1{,}36\ x10^3$	79,5	Sequenced	Sequenced	Sequenced
30/07/2018	PEP 04C	PEP	M	Adult	18,66	$3{,}80\ x10^3$	79,5	+	+	x
31/07/2018	PEP 27	PEP	F	Adult	21,66	$5{,}29x10^2$	79,5	+	+	x
31/07/2018	PEP 28	PEP	M	Adult	23,22	$1{,}90x10^2$	77,5	+	+	x
31/07/2018	PEP 29	PEP	F	Adult	22,1	$3{,}97x10^2$	79,5	Sequenced	Sequenced	Sequenced
31/07/2018	PEP 30	PEP	F	Adult	23,03	$2{,}16x10^2$	79,5	+	+	x
31/07/2018	PEP 03C	PEP	F	Adult	21,37	$6{,}58x10^2$	79,5	+	+	x
31/07/2018	PEP 20B	PEP	F	Adult	22,25	$3{,}63x10^2$	79	-	+	x
01/08/2018	PEP 31	PEP	M	Adult	20,21	$1{,}38\ x10^3$	79,5	+	+	x
01/08/2018	PEP 05C	PEP	M	Adult	22,33	$3{,}43x10^2$	77.5	+	+	x
02/08/2018	PEP 24B	PEP	F	Adult	23,45	$1{,}65x10^2$	79/79,5	+	+	x
02/08/2018	PEP 32	PEP	M	Adult	19,97	$1{,}61x10^3$	79,5	+	+	x
02/08/2018	PEP 33	PEP	M	Adult	23,14	$2{,}06x10^2$	79	+	+	x
03/08/2018	PEP 34	PEP	F	Adult	25,72	$3{,}68\ x10^1$	79	+	+	x
03/08/2018	PEP 35	PEP	F	Adult	Negative	Negative	-	-	-	x
03/08/2018	PEP 36	PEP	F	Subadult	26,48	$1{,}13\ x10^1$	77,5	+	-	x
06/08/2018	PEP 12C	PEP	F	Adult	21,42	$4{,}09x10^2$	79,5	+	+	x
06/08/2018	PEP 37	PEP	F	Adult	16,59	$1{,}26x10^4$	79,5	+	+	x
06/08/2018	PEP 38	PEP	F	Adult	21,61	$3{,}57x10^2$	79,5	+	+	x
07/08/2018	PEP 39	PEP	M	Adult	25,08	$3{,}04\ x10^1$	79	+	-	x
08/08/2018	PEP 40	PEP	F	Adult	24,04	$6{,}38\ x10^1$	79	+	-	x
08/08/2018	PEP 42	PEP	F	Adult	21	$5{,}55x10^2$	79,5	+	+	x
08/08/2018	PEP 01C	PEP	M	Adult	22,03	$2{,}66x10^2$	79,5	+	+	x
09/08/2018	PEP 43	PEP	M	Adult	20,88	$6{,}30x10^2$	79,5	+	+	x
09/08/2018	PEP 26B	PEP	M	Adult	21,54	$3{,}76x10^2$	79	+	+	x
20/08/2018	VBA 10C	VBA	F	Adult	23,88	$7{,}16\ x10^1$	77,5	+	+	x

20/08/2018	VBA 27	VBA	M	Adult	Negative	Negative	-	-	-	x
21/08/2018	VBA 07C	VBA	M	Adult	21,9	$2{,}90x10^{2}$	79,5	+	+	x
24/08/2018	VBA 28	VBA	F	Adult	20,58	$7{,}46x10^{2}$	79,5	+	+	x
24/08/2018	VBA 16B	VBA	F	Adult	26,27	$1{,}31\ x10^{1}$	77,5	+	-	x
24/08/2018	VBA 21B	VBA	M	Puppy	27,46	$5{,}64x10^{0}$	77,5	+	+	x
24/08/2018	VBA 30	VBA	F	Puppy	25,93	$1{,}68\ x10^{1}$	79,5	+	+	x
27/08/2018	VBA 31	VBA	F	Adult	23,29	$1{,}08x10^{2}$	79,5	+	+	x
27/08/2018	VBA 32	VBA	F	Adult	Negative	Negative	-	-	-	x
27/08/2018	VBA 25B	VBA	M	Puppy	Negative	Negative	-	-	-	x
28/08/2018	VBA 33	VBA	F	Adult	25,54	$2{,}32\ x10^{1}$	77,5	+	+	x
28/08/2018	VBA 34	VBA	M	Subadult	22,36	$2{,}17x10^{2}$	77,5	+	+	x
28/08/2018	VBA 35	VBA	M	Subadult	25,55	$2{,}21\ x10^{1}$	79,5	+	+	x
28/08/2018	VBA 36	VBA	M	Puppy	25,29	$2{,}62\ x10^{1}$	76	+	-	x
28/08/2018	VBA 03C	VBA	F	Adult	22,35	$2{,}12x10^{2}$	79,5	Sequenced	Sequenced	Sequenced
29/08/2018	VBA 37	VBA	F	Adult	22,43	$2{,}08x10^{2}$	77,5	+	+	x
30/08/2018	VBA 38	VBA	F	Puppy	27,3	$6{,}33x10^{0}$	79	+	-	x
30/08/2018	VBA 22B	VBA	F	Puppy	27,53	$5{,}39x10^{0}$	79	+	-	x
30/08/2018	VBA 09C	VBA	M	Adult	Negative	Negative	-	-	-	x
31/08/2018	VBA 40	VBA	F	Adult	21,87	$3{,}03x10^{2}$	79,5	+	+	x
01/10/2018	PEP 18B	PEP	F	Puppy	Negative	Negative	-	-	-	x
02/10/2018	PEP 44	PEP	M	Puppy	26,99	$7{,}88x10^{0}$	79,5	+	-	x
02/10/2018	PEP 45	PEP	M	Puppy	20,18	$9{,}85x10^{2}$	79,5	+	+	x
02/10/2018	PEP 46	PEP	F	Puppy	28	$3{,}93x10^{0}$	79	+	-	x
02/10/2018	PEP 47	PEP	M	Puppy	27,04	$7{,}78x10^{0}$	79	+	-	x
03/10/2018	PEP 31B	PEP	M	Adult	18	$4{,}66x10^{3}$	79,5	-	+	x
04/10/2018	PEP 48	PEP	F	Adult	23,54	$9{,}13\ x10^{1}$	77	+	-	x
04/10/2018	PEP 32B	PEP	M	Adult	20,12	$1{,}04x10^{3}$	79,5	+	+	x
04/10/2018	PEP 49	PEP	M	Puppy	Negative	Negative	-	-	-	x
08/10/2018	PEP 01D	PEP	M	Adult	19,93	$1{,}18x10^{3}$	79,5	+	+	x

09/10/2018	PEP 43B	PEP	M	Adult	19,32	1,58x10^{3}	79,5	+	+	x
09/10/2018	PEP 50	PEP	F	Adult	17,55	5,49x10^{3}	79,5	+	+	x
10/10/2018	PEP 51	PEP	M	Adult	17,62	5,22x10^{3}	79,5	+	+	x
22/10/2018	VBA 07D	VBA	M	Adult	21,66	2,96x10^{2}	79,5	+	+	x
23/10/2018	VBA 09D	VBA	M	Adult	Negative	Negative	-	-	-	x
23/10/2018	VBA 11C	VBA	M	Adult	17,19	7,11x10^{3}	79,5	+	+	x
23/10/2018	VBA 03D	VBA	F	Adult	21,45	3,49x10^{2}	79,5	Sequenced	Sequenced	Sequenced
29/10/2018	VBA 21C	VBA	M	Subadult	21,65	3,00x10^{2}	77	+	+	x
31/10/2018	VBA 39B	VBA	F	Adulta	24,13	5,14 x10^{1}	77,5	+	-	x
31/10/2018	VBA 23B	VBA	F	Adulta	27,49	4,74x10^{0}	77,5	+	-	x
31/10/2018	VBA 29B	VBA	M	Puppy	22,09	2,18x10^{2}	79	+	+	x
01/11/2018	VBA 41	VBA	F	Adult	18,94	2,05x10^{3}	79,5	+	+	x
06/11/2018	VBA 42	VBA	M	Puppy	23,49	8,13 x10^{1}	77,5	+	+	x
06/11/2018	VBA 25C	VBA	M	Puppy	25,69	1,70 x10^{1}	79	+	+	x
06/11/2018	VBA 43	VBA	M	Adult	28,35	2,56x10^{0}	79	-	-	x
07/11/2018	VBA 06B	VBA	M	Adult	20,73	5,74x10^{2}	79	+	+	x
07/11/2018	VBA 44	VBA	M	Adult	22,16	2,09x10^{2}	77,5	+	+	x
21/01/2019	VBA 16C	VBA	F	Adult	25,08	2,62 x10^{1}	77,5	+	+	x
21/01/2019	VBA 39C	VBA	F	Adult	Negative	Negative	-	-	-	x
21/01/2019	VBA 41B	VBA	F	Adult	22	2,33x10^{2}	79	+	+	x
22/01/2019	VBA 45	VBA	F	Adult	Negative	Negative	-	-	-	x
22/01/2019	VBA 29C	VBA	M	Subadult	21,02	4,65x102	79,5	+	+	x
22/01/2019	VBA 46	VBA	F	Puppy	20,83	5,33x10^{2}	79,5	+	+	x
23/01/2019	VBA 47	VBA	F	Adult	21,66	2,96x10^{2}	77,5	+	+	x
23/01/2019	VBA 48	VBA	F	Adult	25,55	1,88 x10^{1}	79	+	+	x
23/01/2019	VBA 03E	VBA	F	Adult	22,58	1,55x10^{2}	79,5	+	+	x
23/01/2019	VBA 49	VBA	F	Adult	23,41	1,02x10^{2}	79,5	+	+	x
23/01/2019	VBA 05D	VBA	M	Adult	22,44	1,70x10^{2}	79,5	+	+	x
29/01/2019	VBA 17B	VBA	F	Adult	23,22	9,79 x10^{1}	79,5	+	+	x

29/01/2019	VBA 25D	VBA	M	Juvenil	27,99	3,33x10^{0}	79	+	-	x
29/01/2019	VBA 21D	VBA	M	Juvenil	27,44	4,94x10^{0}	77,5	+	-	x
29/01/2019	VBA 50	VBA	M	Puppy	27	6,72x10^{0}	77,5	+	-	x
30/01/2019	VBA 51	VBA	M	Juvenil	19,46	1,41x10^{3}	77,5	+	+	x
30/01/2019	VBA 52	VBA	F	Juvenil	24,41	4,27 x10^{1}	79,5	+	+	x
20/03/2019	VBA 03F	VBA	F	Adult	24,12	5,39 x10^{1}	79,5	-	+	x
21/03/2019	VBA 21E	VBA	M	Subadult	28,26	2,85x10^{0}	77,5	+	-	x
21/03/2019	VBA 53	VBA	F	Adult	26,13	2,38 x10^{1}	79,5	+	+	x
21/03/2019	VBA 44B	VBA	M	Adult	21,96	2,39x10^{2}	77,5	-	+	x
21/03/2019	VBA 54	VBA	M	Subadult	Negative	Negative	-	+	-	x
22/03/2019	VBA 55	VBA	M	Adult	22,19	2,03x10^{2}	79	+	+	x
25/03/2019	VBA 38B	VBA	F	Subadult	28,05	3,18x10^{0}	79	+	-	x
25/03/2019	VBA 56	VBA	F	Subadult	28,46	2,90x10^{0}	79	+	-	x
25/03/2019	VBA 57	VBA	F	Adult	26,32	1,31 x10^{1}	79	+	-	x
26/03/2019	VBA 16D	VBA	F	Adult	23,26	1,10x10^{2}	77,5	+	+	x
27/03/2019	VBA 25E	VBA	M	Subadult	16,98	9,03x10^{3}	79,5	+	+	x
28/03/2019	VBA 08C	VBA	F	Adult	20,26	8,98x10^{2}	79,5	+	+	x
23/04/2019	VBA 58	VBA	M	Puppy	28,64	2,55x10^{0}	79,5	-	-	x
23/04/2019	VBA 21F	VBA	M	Subadult	14,27	5,98x10^{4}	77,5	+	+	x
23/04/2019	VBA 59	VBA	M	Adult	21,79	3,08x10^{2}	79,5	+	+	x
23/04/2019	VBA 19B	VBA	F	Adult	24,27	5,48 x10^{1}	79,5	+	+	x
25/04/2019	VBA 60	VBA	M	Subadult	22,75	1,58x10^{2}	79,5	+	+	x
26/04/2019	VBA 01B	VBA	M	Adult	26,25	1,36 x10^{1}	79	+	-	x
30/04/2019	VBA 44C	VBA	M	Adult	22,49	1,88 x10^{2}	77,5	+	+	x
30/04/2019	VBA 55B	VBA	M	Adult	22,35	2,09 x10^{2}	79	+	+	x

[1] Parque Estadual do Prosa; [2] Vila da Base Aérea; M: male: F: female; X : not recaptured; +: positive: -: negative
